# DIGITAL INEQUALITY IN OLDER ADULTS’ ONLINE SOCIAL ENGAGEMENT AND SOCIAL CAPITAL

**DOI:** 10.1093/geroni/igz038.3353

**Published:** 2019-11-08

**Authors:** Minh Hao Nguyen, Amanda E Hunsaker, Eszter Hargittai

**Affiliations:** University of Zurich, Zurich, Switzerland

## Abstract

The increasing popularity of social media and other online communities offers new possibilities for older adults to stay socially connected. This study examines the relationship of older adults’ online social engagement and bonding as well as bridging social capital based on a survey of over 1,000 adults aged 60 and over. Social bonding refers to support obtained from existing strong social ties while social bridging is creating connections across varied social networks. We estimated three multi-stage regression models to examine these relationships when controlling for sociodemographic factors, as well as Internet experiences and skills. We then extended the regression models with Internet skills as a moderator. Findings show that older adults who engage more often in specific online social activities (i.e., asking questions on social media, looking at photos of family members/others) enjoy greater bridging social capital (both in offline and online contexts) than those who do so less often. Furthermore, Internet skills moderate the relationship between online social engagement and social capital. Specifically, older adults with greater Internet skills benefit relatively more from engaging in specific online social activities more often with respect to online social bridging. These results imply that digital inequalities may put older adults who are less skilled in using the Internet at a disadvantage when it comes to building social capital from online social engagement. Thus, while social media has potential positive implications for well-being among older adults, the current manifestation of this does not suggest equitable distribution of those benefits across different older users.

